# Composition and Antioxidant, Antienzymatic and Antimicrobial Activities of Volatile Molecules from Spanish *Salvia lavandulifolia* (Vahl) Essential Oils

**DOI:** 10.3390/molecules22081382

**Published:** 2017-08-21

**Authors:** Ana-Belen Cutillas, Alejandro Carrasco, Ramiro Martinez-Gutierrez, Virginia Tomas, Jose Tudela

**Affiliations:** 1 GENZ—Group of Research on Enzymology, Department of Biochemistry and Molecular Biology-A, Regional Campus of International Excellence “Campus Mare Nostrum”, University of Murcia, 30100 Murcia, Spain; alejandro.carrasco@um.es (A.C.); tudelaj@um.es (J.T.); 2 Novozymes Spain S.A., 28224 Madrid, Spain; rma@novozymes.com; 3 Department of Analytical Chemistry, University of Murcia, 30100 Murcia, Spain; virginia@um.es

**Keywords:** *Salvia lavandulifolia*, essential oil, GC-FID, GC-MS, antioxidant capacity, enzymatic inhibition, antimicrobial activity

## Abstract

The current study describes the composition of *Salvia lavandulifolia* (Vahl) essential oils (SlEOs) obtained from plants cultivated in Murcia (Spain), as determined by gas chromatography. Relative and absolute concentrations, the enantiomeric ratios of chiral compounds and the in vitro antioxidant, antienzymatic and antimicrobial activities are described. The main components of the SlEOs were camphor, 1,8-cineole, camphene and α-pinene, and the main enantiomers were (+)-camphor and (−)-camphene. The activities against free radicals and the capacity to reduce and chelate metallic ions were measured. SlEO-3 showed the highest activity in ORAC, DPPH, ABTS and reducing power methods, while SlEO-1 exhibited the highest chelating power. The activity of lipoxygenase and acetylcholinesterase could be inhibited by all the SlEOs, being bornyl acetate and limonene the most active individual compounds against lipoxygenase and 1,8-cineole against acetylcholinesterase. SlEOs and some individual compounds inhibited *Escherichia coli*, *Staphylococcus aureus* and *Candida albicans*. These results increase our knowledge of SlEOs and, particularly, provide for the first time a complete characterization of SlEOs from Murcia, Spain, while proposing possible biotechnological uses for them.

## 1. Introduction

*Salvia*, the largest genus of the Lamiaceae family, comprises nearly 900 species distributed throughout the world. Some species of *Salvia* have been cultivated for their use in folk medicine and for culinary purposes. *Salvia lavandulifolia* Vahl. (*Salvia officinalis* subsp. *lavandulifolia* (Vahl) Gams or Spanish sage) is native to the Iberian Peninsula and grows from south-east of Morocco to the Mediterranean area of France. It is a small woody herbaceous perennial shrub, ranging from 17 to 100 cm high, with mauve-blue flowers that usually grows on the sandy-calcareous soils of mountainous areas (from 300 to more than 1000 m above sea level) [1].

The essential oil (EO) composition of the genus *Salvia* is highly variable, depending on climatic, geographical and seasonal conditions as well as genotypic factors [2,3,4,5]. *S. lavandulifolia* EO (SlEO) has been described as a safe memory enhancer, suggesting it could be used in Alzheimer’s disease therapy [6,7,8,9]. Moreover, SlEO presents other notable activities with medical applications, which include spasmolitic [10], oestrogenic [11] and antimicrobial [12] properties.

The most common technique used for the analysis of volatile components is gas chromatography coupled with a flame ionization detector (GC-FID) or with a mass spectrometry detector (GC-MS), because these methods provide qualitative and quantitative data, even for complex mixtures, with high sensitivity and resolution. Some studies have reported the relative quantitation of SlEO from other Spanish regions [1,7,13,14,15,16] and from other countries (Poland [17], Germany [12] and Brazil [18,19]). None of them have studied SlEOs from Murcia nor made a chiral characterization of the EOs from this species. The chiral distribution of the principal compounds, as an essential part of quality assurance, helps to determine the genuineness or adulteration of EOs [20]. Besides, the enantiomeric ratio of a compound can cause significant differences from the point of view of sensorial analysis and bioactivity [21,22].

Reactive oxygen species (superoxide radical, hydrogen peroxide, etc.) have been implicated in the pathology of several human diseases (cancer, atherosclerosis, malaria, rheumatoid arthritis and neurodegenerative diseases, and aging effects [23,24,25,26,27]), and in the deterioration of food and cosmetic products [28]. The use of EOs as natural antioxidant is regarded as a safe alternative to synthetic ones [29,30].

Lipoxygenase enzyme (LOX) produces hydroperoxyeicosatetraenoic acids (HPETEs) and hydroxyoctadecadienoic acids (HODEs) from arachidonic acid and linoleic acid, respectively [31]. SlEO can be useful to inhibit this enzyme and, therefore, may represent a valid strategy for prevention and therapy of Alzheimer’s, cancer and inflammatory diseases [32]. As well as this bioactivity, SlEO shows acetylcholinesterase (AChE) inhibitory activity [6,9,11,33], so it can be used as memory enhancer in Alzheimer’s disease [34,35].

Some studies from other regions or countries reported the antibacterial and antifungal activities of EOs from *Salvia* genus [19,36,37,38]. These activities are highly related with the particular EO composition and microbial strain used in the assays. The antimicrobial capacity of EOs lends weight to their use as natural preservatives.

In this study, we describe the detailed volatile compositions of four SlEOs from the province of Murcia (Spain) and evaluate whether they fulfill the requirements of ISO 3526 [39]. The enantiomeric ratios of the commercially available compounds are also reported. The in vitro antioxidant and antimicrobial activities of these SlEOs are studied to ascertain their potential use as natural preservatives. The inhibition of LOX and AChE by SlEOs could lead to useful strategies for the treatment of inflammatory and Alzheimer’s diseases and for natural insecticidal purposes.

## 2. Results and Discussion

### 2.1. Fast Gas Chromatography (FGC-FID and FGC-MS) Study

#### 2.1.1. Experimental Data

The obtained yields for all the SlEOs ranged from 0.8% to 1.2% (*v*/*w*). The identified volatile components in the four SlEOs are summarized in Table 1, where >99% of the total was identified, and >93% of the total area is also expressed in absolute concentration. All four SlEOs under study contain camphor (30.8–37.2%), 1,8-cineole (21.7–25.7%), camphene (7.2–9.4%) and α-pinene (4.8–5.5%) as main constituents. β-Pinene (4.0–5.6%), limonene (2.8–4.4%), myrcene (1.3–1.6%) and sabinene (1.3–1.8%) had significant concentrations in all the SlEOs.

To our knowledge, none of the previous studies have calculated the absolute amount of the volatile compounds. A higher percentage does not always mean a larger concentration; for instance, in this study, SlEO-1 has a higher percentage of 1,8-cineole than SlEO-3, but the concentration in the first is lower. Moreover, this characterization is useful to detect EO adulterations with organic solvents.

**Figure 1:**
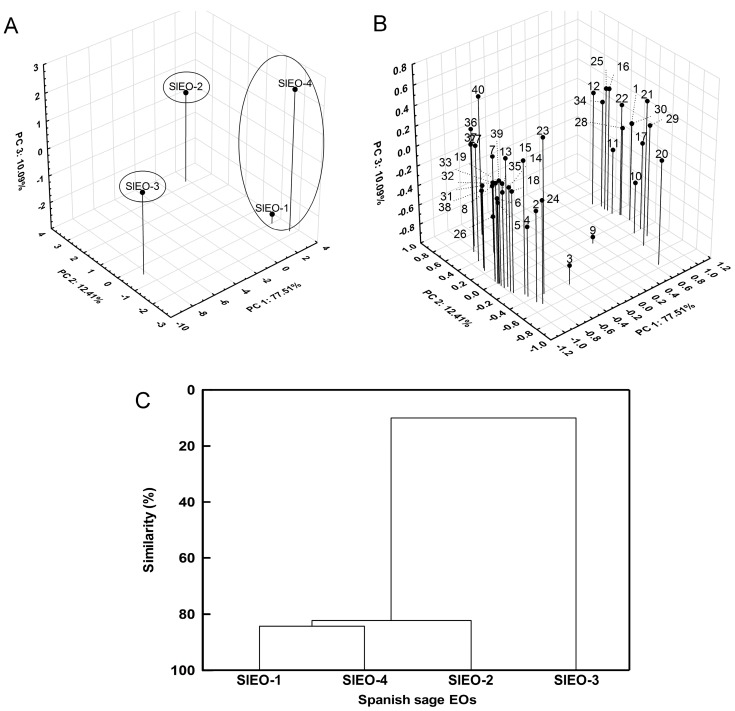
PCA and AHC analyses. (**A**) 3D-score plot of PC3 vs. PC2 and PC1; (**B**) 3D-loading plot of PC3 vs. PC2 and PC1; (**C**) AHC dendrogram: percentages of similarity between studied EOs and clusters.

Principal component analysis (PCA) was carried out with the relative compositions of the SlEOs and determined three principal components (Figure 1A,B): PC1 (77.51% of the total variance), PC2 (12.41% of the total variance) and PC3 (10.09% of the total variance). SlEO-1, -2 and -4 have similar values for PC1, which contributes to the highest percentage of the total variance.

Forty different compounds were described in the SlEOs (Figure S1). The loading plot (Figure 1B) allows us to establish relations between composition and the differences exhibited in the score plot. SlEO-1, -2 and -4 have similar concentrations of linalool (**16**), *cis*-sabinol (**17**), α-terpineol (**22**), linalyl acetate (**25**), sabinyl acetate (**28**) and geranyl propionate (**34**), among others. However, SlEO-3 showed lower concentrations of the compounds detailed above, but higher concentrations of camphor (**18**), borneol (**19**), β-caryophyllene (**32**), α-humulene (**33**) and α-curcumene (**35**).

Using the relative area of all compounds of the SlEOs (Table 1), the agglomerative hierarchical clustering (AHC) based on Euclidean distance was represented in a dendrogram (Figure 1C), showing that SlEO-1 and -4 are the most similar with 84.2% similarity, followed by SlEO-2 with 82.3% similarity. This cluster of SlEOs reveals 9.8% of similarity with SlEO-3.

#### 2.1.2. Comparison with Previous Studies from Other Regions and Countries

Other studies have reported the relative composition of SlEOs. Some differences existed in the main compounds; for example, while 1,8-cineole is present in our SlEOs with a maximum of 25.7%, in other studies from Central Spain, this percentage reached 54.7% of the total area [40]. Also, camphor showed high variability, ranging from 0% to 29% of the EO depending on the origin [1,7].

Most studies concerning the composition of SlEOs have been carried out in other regions of Spain. The EO composition depends on various external factors [13,14,16,40]. For example, 1,8-cineole and camphor were abundant components in almost all studies, while α-pinene, β-pinene, limonene and linalool were considered the major components in fewer cases [1,14,15]. Our SlEOs from Murcia and those analyzed in other studies (except the one reported by Porres-Martinez et al. [13]) described the presence of β-myrcene in different percentages. This compound has been regarded as a chemotaxonomic marker of SlEO since it is relatively rare in other sage species [41]. Some SlEOs have high amounts of β-phellandrene [13] and spathulenol [1,40], whereas they are not present in others. These molecules can be used as markers of their respective growing areas.

Using the identified compounds (Table 1), oxygenated monoterpenes were clearly predominant (66.8–73.2% of the total area) and oxygenated sesquiterpenes represented the lowest percentage (0.4–0.5% of the total area). The most abundant organic functional group was ketone (30.8–37.2% of the total area), followed by the ether group (22.1–26.1% of the total area). This distribution is also found in other studies [13,14,15]. Herraiz-Penalver et al. [1] reported EO compositions of 20 SlEOs from the central region of Spain, which presented a higher percentage of monoterpenes than sesquiterpenes, with hydrocarbon monoterpenes as the major fraction.

The International Organization for Standardization (ISO) has published International Standards for SlEO [39]. Most of our results match the requirements of the ISO standard, although all the SlEOs showed a higher percentage of 1,8-cineole and camphor and SlEO-3 had a slightly lower amount of linalyl acetate, α-terpinyl acetate and sabinyl acetate (Table 2). Some SlEOs from other regions of Spain fell below the requirements in the case of limonene [14], camphor [1,16], borneol [13] and sabinyl acetate [1,13,15,16] or exceeded the requirements for α-pinene [1], 1,8-cineole [1,16], linalool [15], and borneol [7].

### 2.2. Enantioselective Gas Chromatography-Mass Spectrometry (EsGC-MS) Study

The enantiomeric ratios of commercially available components of SlEOs from Murcia have been obtained (Table 3). The (+)-enantiomer prevails in the case of limonene, sabinene hydrate, camphor, terpinen-4-ol, α-terpineol and α-terpinyl acetate, while the (−)-enantiomer predominates in camphene, β-pinene, linalool, bornyl acetate, borneol and β-caryophyllene. Linalool and α-terpineol in SlEO-1 and limonene and terpinen-4-ol in SlEO-3 showed different distribution percentages compared with the other SlEOs. Different enantiomeric distributions of chiral compounds could provide diverse organoleptic properties, information about the origin, and also act as a tool to detect possible fraud in commercial EOs. To our knowledge, this study is the first one to present an extensive enantiomeric characterization of SlEO.

### 2.3. Antioxidant Capacity

#### 2.3.1. ORAC

The antioxidant activities of the four SlEOs were as follows (Table 4): SlEO-3^ORAC^ > SlEO-2^ORAC^ ≈ SlEO-4^ORAC^ > SlEO-1^ORAC^. ORAC values were expressed in mg Trolox equivalents (TE)/g SlEO, using the Trolox Equivalent Antioxidant Capacity (TEAC) units. The two main components, camphor and 1,8-cineole, did not show any ORAC antioxidant activity, so that any antioxidant potential of these SlEOs is due to the minor compounds they contain. SlEO-3 has more β-caryophyllene than the other SlEOs. This compound is one of the most active as seen by this method (Table 5). Interestingly, SlEO-1 has lower amounts of linalool and linalyl acetate than SlEO-2 and -4, and these compounds, which showed antioxidant activity in this method, could be responsible for the difference.

#### 2.3.2. DPPH

This method showed the following results (mg TE/g SlEO) (Table 4): SlEO-3^DPPH^ > SlEO-4^DPPH^ ≥ SlEO-2^DPPH^ ≥ SlEO-1^DPPH^. All SlEOs were moderately effective in this method, because only two compounds, α-terpinene and γ-terpinene, showed activity against this radical (Table 5).

#### 2.3.3. ABTS

The activity against ABTS radical was as follows (mg TE/g SlEO) (Table 4): SlEO-4^ABTS^ ≈ SlEO-3^ABTS^ > SlEO-1^ABTS^ ≈ SlEO-2^ABTS^. α-Terpinene and γ-terpinene are the most active compounds tested in this assay (Table 5).

#### 2.3.4. Reducing Power (RdP)

This method showed the following results expressed in mg ascorbic acid equivalents (AAE)/kg SlEO (Table 4): SlEO-3^RdP^ > SlEO-2^RdP^ > SlEO-1^RdP^ > SlEO-4^RdP^. SlEO-3 showed the highest amount of camphene and SlEO-4 has slightly less amount of α-terpinene and γ-terpinene. 

#### 2.3.5. Chelating Power (ChP)

The result from this method was expressed in mg ethylenediaminetetraacetic acid equivalents (EDTAE)/g SlEO (Table 4): SlEO-1^ChP^ > SlEO-4^ChP^ ≥ SlEO-3^ChP^ ≥ SlEO-2^ChP^. The highest activities of individual compounds to chelate Fe^2+^ ion were showed by linalool > α-terpinene > linalyl acetate > *p*-cymene (Table 5).

### 2.4. Inhibitory Activity

#### 2.4.1. LOX Inhibition

The degree of inhibition (DI) of LOX was measured at 150 µg/mL for all SlEOs. The results were the following: SlEO-4^LOX^ (42.4 ± 0.7%) ^a^ > SlEO-2^LOX^ (40.4 ± 0.8%) ^b^ > SlEO-1^LOX^ (36.6 ± 0.5%) ^c^ > SlEO-3^LOX^ (34.2 ± 0.7%) ^d^. In addition, ${\mathrm{IC}}_{50}^{\mathrm{LOX}}$ of some individual compounds has been measured: Bornyl acetate (74.5 ± 2.8 µg/mL), limonene (116.1 ± 3.3 µg/mL), camphor (417.7 ± 13.0 µg/mL) and linalool (516.0 ± 6.8 µg/mL). Compounds that could not reach 50% inhibition are expressed as DI (%) at 514.2 µg/mL: 1,8-cineole (30.9 ± 1.1%), terpinen-4-ol (29.6 ± 1.0%) and α-terpineol (17.4 ± 0.2%). The ${\mathrm{IC}}_{50}^{\mathrm{LOX}}$ obtained with NDGA (102.6 ± 2.8 μg/mL) was similar to previous studies [42].

SlEO-1, -2 and -4 showed high amounts of limonene and linalool. Bornyl acetate and limonene were the most active LOX inhibitors. Previous studies showed the anti-LOX activity of other *Salvia* species. Higher IC_50_ was showed by the EO from *S. officinalis* with a 59% of 1,8-cineole [43]. However, studies with *Salvia* species from southern Africa showed lower IC_50_ values [44,45]. Although those EOs were obtained from *Salvia* genus, the composition of those EOs is different from that of the EOs analyzed in this study, therefore, the results can be significantly different. Studies carried out with *Rosmarinus officinalis* EO (high content of 1,8-cineole, camphor, α-pinene, borneol and camphene) [46] showed anti-LOX capacity similar to that shown with the EOs of this study. No previous studies have been found that analyze the ability of SlEOs to inhibit LOX.

#### 2.4.2. AChE Inhibition

SlEOs showed the following ${\mathrm{IC}}_{50}^{\mathrm{AChE}}$ (µg/mL): SlEO-4^AChE^ (108.0 ± 4.2) ^b^ ≈ SlEO-1^AChE^ (108.9 ± 3.5) ^b^ ≈ SlEO-2^AChE^ (119.3 ± 10.4) ^b^ < SlEO-3^AChE^ (142.4 ± 6.1) ^a^. In relation to individual compounds, 1,8-cineole (${\mathrm{IC}}_{50}^{\mathrm{AChE}}$ = 35.2 ± 1.5 µg/mL), was the most potent AChE inhibitor. α-Pinene also showed anticholinesterase activity (${\mathrm{IC}}_{50}^{\mathrm{AChE}}$ = 446.1 ± 7.9 µg/mL). Camphene and terpinen-4-ol could inhibit the enzyme, but it was not possible to calculate their ${\mathrm{IC}}_{50}^{\mathrm{AChE}}$ due to their low water solubilities (15% inhibition reached at 42.4 ± 3.7 µg/mL for camphene and at 582.4 ± 38.1 µg/mL for terpinen-4-ol). Other compounds present in these SlEOs showed no inhibition activity or could not be tested due to their limits of solubility. Galantamine was used as standard inhibitor (${\mathrm{IC}}_{50}^{\mathrm{AChE}}$ = 0.16 ± 0.03 µg/mL).

Previous studies [8,47,48] reported bovine and human erythrocyte anticholinesterase activities of commercial SlEOs and their relevant compounds, with some differences compared with our results concerning *Electrophorus electricus* acetylcholinesterase, perhaps due to the different enzymes studied. Arruda, et al. [49] reported higher IC_50_ values of anticholinesterase activity of EOs from *Hedychium gardnerianum* using acetylcholinestase from *E. electricus*. However, Bonesi, et al. [50] described similar or lower IC_50_ values than those reported in this study using the EOs from three *Pinus* species. These differences are mainly due to different composition of EOs. Generally, EOs showed lower anticholinesterase capacities than galantamine and other alkaloids used in the treatment of Alzheimer’s disease, but EOs are highly volatile, allowing their use in aromatherapy, which could reduce the side effects [49].

### 2.5. Determination of Minimum Inhibitory Concentration (MIC) and Minimum Bactericidal (MBC) or Fungicidal (MFC) Concentrations

The SlEOs and most compounds with relative concentrations higher than 0.5% were tested against *P. aeruginosa*, *E. coli*, *S. aureus* and *C. albicans*. MIC values for *P. aeruginosa* (Gram-negative) were higher than 20 µL/mL with all the SlEOs tested (data not shown). The results (Table 6) show that Gram-positive bacteria are more sensitive to the EOs than Gram-negative ones, in agreement with other studies [5]. α-Pinene and *p*-cymene were more effective against *E. coli* than *S. aureus*, whereas limonene, linalool, borneol, terpinen-4-ol and α-terpineol were more active against the latter. *C. albicans* was inhibited by α-pinene, *p*-cymene, limonene and borneol. The results for all the SlEOs were similar to each other because the concentrations of their active molecules were similar. In the case of SlEO-3 (the most different one), it had a lower amount of limonene and linalool, but higher content of borneol, thus compensating for the overall result. The SlEOs showed bactericidal effects because the MBC/MIC was lower than 4, reflecting other studies in different plant species [36,51]. The MIC and MBC values obtained in this study were higher [38,52,53], similar [19,54,55] or lower [56,57] than those reported in previous studies with EOs from *Salvia*. The lipophilic character of essential oils allows them to pass through cell membranes and produce the lysis [58]. Although the antimicrobial activity of EOs is weaker than that produced by reference antimicrobials [52], they could contribute to the preservation of cosmetic and food products where EOs are mainly used for their odorant characteristics.

## 3. Materials and Methods

### 3.1. Essential Oils

SlEOs were obtained from 300 g of the aerial part of plants grown in the south-east of Spain by means of hydrodistillation for 3 h in a Clevenger-type apparatus [59], dried over anhydrous sodium sulfate and stored at 4 °C until use. SlEO-1 and -4 were obtained from plants grown in the Lower Meso-Mediterranean bioclimatic zone, SlEO-2 from plants grown in the Upper Meso-Mediterranean bioclimatic zone and SlEO-3 from plants grown in the Supra-Mediterranean bioclimatic zone [60]. Plant species were identified in the Plant Biology Department of Murcia University by Dr. Pedro Sanchez-Gomez. The Department of Biochemistry and Molecular Biology-A stores the voucher specimens (BMBA160611, BMBA160612, BMBA160613, BMBA160614 for SlEO-1, -2, -3 and -4, respectively).

### 3.2. Reagents and Solvents

The chemical compounds used for the antioxidant methods, the reagents for LOX and AChE inhibition assays and reference antibacterial and antifungal compounds were purchased from Sigma-Aldrich (Madrid, Spain). All compounds were of analytical grade (purity higher than 95%). All culture media were acquired from VWR Chemicals (Barcelona, Spain): Mueller-Hinton agar (MHA), Mueller-Hinton broth (MHB), Roswell Park Memorial Institute medium (RPMI-1640), Sabouraud dextrose agar (SDA), tryptic soy broth (TSB) and yeast peptone dextrose (YPD). Solvents of analytic grade and buffers were purchased from Merck (Madrid, Spain). Type I (18 MΩ·cm) deionized water (MilliQ-Reference, Millipore, Madrid, Spain) was used in this work.

### 3.3. FGC-FID and FGC-MS

The relative proportions of the SlEO compounds were determined using a GC7890 chromatograph (Agilent, Madrid, Spain) equipped with a FID. The detector temperature was 320 °C. The sandwich injections (0.2 µL air, 0.2 µL isooctane, 0.2 µL air, 0.3 µL sample and 0.2 µL air, described from plunger to needle) were made using a MPS-2XT automatic multi-purpose sampler (Gerstel, Sevilla, Spain).

The chromatography was performed in a low bleed capillary fused-silica column, SLB-5ms from Supelco (Madrid, Spain; 15 m length × 0.1 mm internal diameter × 0.1 µm film thickness) with hydrogen as carrier gas (0.8 mL/min) which generates a head pressure of 46.345 psi. This carrier gas was produced with an electrolytic Parker-Domnik-Hunter (Barcelona, Spain) generator.

The injection conditions were as follows: septum purge 3 mL/min, split ratio 100:1 and injector temperature 300 °C. GC oven temperature was kept at 60 °C and programmed to raise up to 300 °C as follows: to 92 °C at a rate 15 °C/min, to 96 °C at a rate of 1 °C/min, to 108 °C at a rate of 20 °C/min and kept constant for 0.5 min, to 120 °C at a rate of 5 °C/min, to 160 °C at a rate of 20 °C/min, to 170 °C at a rate of 5 °C/min and to 300 °C at a rate of 30 °C/min, kept constant at 300 °C for 0.5 min.

The absolute concentrations were determined using calibration curves of each commercially available component described in the SlEOs (Table S1), using the gas chromatograph previously described, coupled with an Agilent MS5975 mass spectrometer with electronic impact ionization and single quadrupole. MS was adjusted to the following conditions: electron ionization energy 70 eV, electron-multiplier voltage 1129, acquisition mass range 30–300 *m*/*z*, 21.035 scans·s^−1^, transfer line temperature 280 °C, ion source temperature 230 °C, MS quadrupole temperature 150 °C.

Compounds were identified by comparison of their retention times and the mass spectra of commercially available pure standards (Figures S2 and S3) and the NIST 08 and Wiley 7 data bases. Several dilutions of SlEOs have been made in 2,2,4-trimethylpentane, to obtain their relative and absolute determinations.

### 3.4. EsGC-MS

A Chiraldex B-DM column (Astec, Madrid, Spain; 30 m length × 0.25 mm internal diameter × 0.12 µm film thickness) from Supelco, made of 2,3-di-*O*-methyl-6-t-butylsilyl β-cyclodextrin, non-bonded to fused silica column, was installed in the previously described device. The injections were similar to the one previously described but, in this case, 0.5 μL of sample was injected. The injector and transfer line temperatures were 200 °C. Hydrogen was used as carrier gas (constant flow of 2.5 mL/min, 8 psi starting column head pressure). The column temperature was programmed to increase from 35 °C to 170 °C at a rate of 4 °C/min.

To identify both enantiomers, the retention times and the mass spectra of commercially available pure standards were compared with those of the SlEO compounds, and confirmed with the NIST and Wiley spectral data bases (Figure S4).

### 3.5. Antioxidant Capacity

Five antioxidant methods were performed with SlEOs and their main individual compounds in triplicate. In the oxygen radical absorbance capacity (ORAC) assay, peroxyl radicals are produced by 2,2′-Azobis(2-methylpropionamidine) dihydrochloride (AAPH), which reacts with fluorescein giving a non-fluorescent compound [61]. The antioxidants are able to scavenge these peroxyl radicals. Trolox was used as the reference antioxidant. The 2,2-diphenyl-1-picrylhydrazyl (DPPH) [62] and 2,2′-azino-bis(3-ethylbenzothiazoline-6-sulphonic acid) (ABTS) [63,64] methods measure the bleaching produced by the reduction of the coloured DPPH and ABTS radicals. Trolox was used as the reference antioxidant. The RdP method measure the ability to reduce ferric ions, according to the method of Oyaizu [65]. Ascorbic acid was the reference antioxidant used in this method. The ChP method measured the ability to chelate Fe^2+^ ion, following the method of Miguel et al. [66]. EDTA was used as reference antioxidant. The SlEOs were diluted in 100% ethanol in the DPPH, ABTS, RdP and ChP methods, whereas in the ORAC method, SlEOs were diluted in 15% ethanol to obtain a final concentration of 5% ethanol in the reaction medium. All measurements were made at the end-point of the reaction, except in the ORAC method (Figure S5).

### 3.6. Inhibition Activity

A LOX preparation from *Glycine max* (soybean) was acquired from Sigma-Aldrich. LOX inhibitory activity was determined as previously reported [67], based on the absorption at 234 nm of the hydroperoxyde conjugated dienes (ε = 25,000 M^−1^ cm^−1^), which are generated from the oxidation of linoleic acid in the presence of oxygen and LOX. This assay was carried out on a double beam Lambda 35 spectrophotometer (PerkinElmer, Madrid, Spain) with the UV-Winlab software, at 25 °C. SlEOs were diluted in 10% acetonitrile (supergradient quality, A_210_ < 0.018) and nordihydroguaiaretic acid (NDGA) was used as standard LOX inhibitor.

AChE VI-S from *Electrophorus electricus* was purchased from Sigma-Aldrich. AChE inhibitory activity was measured in triplicate using a 96-well microplate reader at 25 °C, according to Ellman’s method [68]. This enzyme hydrolyzes acetylthiocholine to acetate and thiocholine, which reacts with 5,5-dithio-bis-(2-nitrobenzoic acid) (DTNB) producing a coloured compound with absorbance at 412 nm. The reaction was measured for 10 min at 25 °C, using a 96-well microplate reader. SlEOs were diluted in 15% ethanol, to obtain a final concentration of 5% ethanol in the reaction medium. Galantamine hydrobromide was used as reference AChE inhibitor.

The DI in both antienzymatic assays were calculated using Equation (1):

(1) $$DI\;\left(\%\right)=\;\frac{\nu_{0}-\nu_{i}}{\nu_{0}}\;\times \;100$$

where $v_{0}$ and $v_{1}$ are the steady state rates without and with inhibitor, respectively. The inhibitions of LOX were reported as DI at 150 µg/mL of each SlEO. Higher SlEO concentrations could not be assayed due to their solubility limits. However, AChE inhibition could be expressed as IC_50_. To calculate the IC_50_ values, data of DI (%) of seven different concentrations of each SlEO were plotted and fitted by non-linear regression according to Equation (2) using Sigma Plot software (systatsoftware.com) (Figure S6). The inhibitions of LOX and AChE by individual compounds were reported as IC_50_ or DI, depending on their inhibition capacities and solubilities.

(2) $$DI\;\left(\%\right)=\;\frac{DI_{max}\;{\left[I\right]}_{0}}{IC_{50}+\;{\left[I\right]}_{0}}$$

### 3.7. Antimicrobial Activity

#### 3.7.1. Microorganisms and Culture Conditions

The following test microorganisms used in this work were acquired from Sigma-Aldrich: *Pseudomonas aeruginosa* ATCC 9027, *Escherichia coli* ATCC 8739, *Staphylococcus aureus* ATCC 6538, and *Candida albicans* ATCC 10231. The stock cultures were preserved in TSB or YPD with 15% glycerol, for bacteria and yeast cells, respectively, at −80 °C. Isolated colonies from an 18- to 24-h agar plate were transferred to MHB for bacteria and RPMI-1640 for *C. albicans*.

#### 3.7.2. MIC and MBC or MFC

MIC were determined using the broth microdilution method in 96-well microplates, according to the M07-A10 [69] standard for bacteria and the M27-A3 [70] for *Candida*. Briefly, two-fold dilutions of a 40 µL/mL SlEOs were prepared to obtain a final concentration range of 0.08–20 µL/mL with 0.5% Tween^®^80 and 2.5% DMSO. The antimicrobial activities of main compounds were also tested in the concentration range of 0.12–15 mM with 0.5% Tween^®^80 and 2.5% DMSO as solvent. The final strain concentration was 5 × 10^5^ CFU/mL in MHB for the bacteria and 0.5−2.5 × 10^3^ in RPMI-1640 for the yeast. These plates were incubated for 24 h for bacteria and 48 h for *Candida*, both at 35 ± 1 °C, under aerobic conditions on a plate shaker at 100 rpm. Streptomycin (0.06–8.00 µg/mL) and fluconazole (0.13–16 µg/mL) were used as reference antibacterial and antifungal compound, respectively. The negative and positive controls were made to test that all solutions were sterile and that 0.5% tween and 2.5% DMSO, used for emulsifying the SlEOs, did not show any antimicrobial activity. MIC is defined as the lowest concentration of EO or individual compound with no visible growth of microorganisms, at the end of the incubation period. Then, 100 µL of each well without growth in the MIC assay were spread on MHA (bacteria) or SDA (yeast) and incubated for 24 h at 35 ± 1 °C to determinate the MBC or MFC. The MBC or MFC was defined as the lowest EO concentration in which microorganisms failed to grow in broth and on agar. All determinations were carried out in triplicate.

### 3.8. Statistical Analysis

The statistical analyses of data were made using both univariate and multivariate methods [71] Data were recorded as mean ± standard deviation of at least triplicate determinations. Data values of 0.0 in the tables means values lower than 0.05 units. Data quality was analyzed by ANOVA, and means were confronted using Tukey’s (HSD) test, considering differences to be significant at *p* < 0.05, represented by different letters next to numerical values in the text and tables. To determine similarity between SlEOs, PCA and AHC based on Euclidean distance were performed. Statistica software (software.dell.com) was used to conduct all statistical analyses.

## 4. Conclusions

*S. lavandulifolia* has been widely used for its medicinal and flavoring properties [7]. The composition of its EO can vary depending on several factors [14]. In this study, four SlEOs obtained from plants grown in Murcia were soundly analyzed. They were rich in α-pinene, camphene, 1,8-cineole and camphor. Moreover, other compounds were found in significant amounts, such as β-pinene, sabinene, myrcene, limonene and borneol. PCA and AHC analyses showed that SlEO-1, -2 and -4 are very similar, but different from SlEO-3. SlEO-1, -2 and -4 have similar concentrations of linalool (**16**), *cis*-sabinol (**17**), linalyl acetate (**25**) and sabinyl acetate (**28**), whereas SlEO-3 has higher concentration of camphor (**18**), borneol (**19**), (−)-myrtenol (**23**), (*Z*)-citral (**24**) and others.

The enantiomeric distribution for sabinene hydrate, camphor, bornyl acetate, borneol and β-caryophyllene were similar in all SlEOs, so they can be used as biomolecular markers for these EOs. The enantiomeric distribution is important to prove the authenticity of EOs.

As regards the antioxidant results, SlEO-3 was the most antioxidant in ORAC, DPPH, ABTS and RdP methods. These differences between methods can be explained by the different antioxidant activities of their individual compounds in each method; for example, linalool has a high ChP, but it is less active than others against ABTS radical.

All the SlEOs showed LOX inhibition at 150 µg/mL. Although bornyl acetate and limonene were the most active against LOX, the two main compounds (camphor and 1,8-cineole) were also able to reduce the rate of the LOX reaction. Moreover, SlEOs are effective against AChE activity. 1,8-Cineole, a major compound in these EOs, is able to reach 50% inhibition at a concentration of 35 µg/mL. These SlEOs could be used for memory enhancement due to their anti-AChE activities and their low proportion of α-thujone [6,9,33]. In fact, there is negligible quantities of α-thujone in SlEOs from Murcia, that prevent their undesirable effects on brain, muscle, kidney and liver cells [58].

The SlEOs of this study also showed antimicrobial activity against *E. coli*, *S. aureus* and *C. albicans*, but not against *P. aeruginosa*, which is a highly resistant Gram-negative bacterium. *S. aureus* and *C. albicans* showed greater susceptibility to the SlEOs than *E. coli*, probably because of some individual components, such as limonene and borneol, which are more effective against these two microorganisms.

The extensive knowledge acquired about the composition and the antioxidant, antienzymatic and antimicrobial activities of these SlEOs is useful for confirming their potential applications in agronomic, food, cosmetic and pharmaceutical industries.

## Figures and Tables

**Table 1:** Fast gas chromatography determination of SlEO compounds.

N ^a^	LRI ^b^	LRI ^c^	Compound	Qualifying and Quantitation Ions ^d^ (*m*/*z*)	SlEO-1		SlEO-2		SlEO-3		SlEO-4		IM
					Concentration	Area	Concentration	Area	Concentration	Area	Concentration	Area	
					(mmol/L ± SD)	(% ± SD)	(mmol/L ± SD)	(% ± SD)	(mmol/L ± SD)	(% ± SD)	(mmol/L ± SD)	(% ± SD)	
1	922	923	Tricyclene	79, 93, 121, 136		0.1f ± 0		0.1g ± 0				0.1e ± 0	1,2
2	928	926	α-Thujene	77, 91, 93, 136		0.5f ± 0		0.4f ± 0		0.6e ± 0		0.5f ± 0.1	1,2
3	930	936	α-Pinene	77, 91, **93**, 121	347.0b ± 7.6	5.5e ± 0	297.2c ± 3.4	4.8g ± 0	382.8a ± 9.3	5.3f ± 0	322.7bc ± 15.4	4.9g ± 0	1,2,3
4	943	953	Camphene	79, **93**, 121, 136	582.1b ± 9.9	8.1f ± 0	491.0c ± 3.3	7.2h ± 0	759.3a ± 21.1	9.4e ± 0	559.2b ± 42.4	7.5g ± 0	1,2,3
5	964	974	Sabinene	77, 91, **93**, 136	100.0c ± 1.7	1.4f ± 0	105.0b ± 0.9	1.4f ± 0	130.4a ± 1.1	1.8e ± 0	92.6d ± 2	1.3g ± 0	1,2,3
6	970	982	β-Pinene	69, 79, 91, **93**	267.7c ± 2.8	4.3f ± 0	281.4b ± 2.3	4.3.f ± 0	416.4a ± 2.8	5.6e ± 0	249.1d ± 3.8	4.0g ± 0	1,2,3
7	979	989	Myrcene	41, **69**, 93, 121	115.0c ± 1.2	1.3g ± 0	120.6b ± 1	1.4f ± 0	135.5a ± 0.6	1.6e ± 0	104.4d ± 2.5	1.3f ± 0	1,2,3
8	999	1008	α-Phellandrene	77, **93**, 119, 136	2.7a ± 0.1	tr	1.7c ± 0	tr	2.0b ± 0.2	tr			1,2,3
9	1008	1017	α-Terpinene	91, **93**, 119, 121	5.6a ± 0.2	0.1 ± 0	3.4b ± 0	tr	3.1c ± 0.1	tr	2.8c ± 0.1	tr	1,2,3
10	1011	1024	*p*-Cymene	91, 117, **119**, 121	31.4b ± 0.3	0.8e ± 0	32.0b ± 0.4	0.7g ± 0	28.5c ± 0.2	0.6h ± 0	33.3a ± 0.7	0.7f ± 0	1,2,3
11	1020	1028	Limonene	67, **68**, 79, 93	162.6a ± 4.1	4.4e ± 0	162.5a ± 1.6	4.2f ± 0	106.7c ± 1.4	2.8h ± 0	144.3b ± 2.6	3.8g ± 0	1,2,3
12	1023	1032	1,8-Cineole	43, 81, **93**, 108	1303.6a ± 55.6	24.2g ± 0.1	1362.6a ± 35.2	25.7e ± 0.1	1326.1a ± 20.5	21.7h ± 0.1	1375.2a ± 207.8	24.7f ± 0.1	1,2,3
13	1053	1055	γ-Terpinene	77, 91, **93**, 119	14.3a ± 0.5	0.1h ± 0	6.7c ± 0.1	0.1g ± 0	8.1b ± 0.4	0.2e ± 0	5.8c ± 0.1	0.1f ± 0	1,2,3
14	1070	1071	Sabinene hydrate	77, 91, **93**, 121	14.9c ± 0.6	0.2f ± 0	14.4c ± 0.5	0.2f ± 0	30.7a ± 0.5	0.4e ± 0	18.9b ± 0.7	0.2f ± 0	1,2,3
15	1080	1086	Terpinolene	91, **93**, 121, 136	10.8a ± 0.3	0.2h ± 0	7.9b ± 0.4	0.2g ± 0	11.2a ± 0.3	0.3e ± 0	6.5c ± 0.4	0.2f ± 0	1,2,3
16	1081	1105	Linalool	41, 67, **69**, 93	71.1c ± 1.1	1.3g ± 0	97.7a ± 2.1	1.8e ± 0	10.0d ± 0.3	0.2h ± 0	90.0b ± 4	1.7f ± 0	1,2,3
17	1143	1143	*cis*-Sabinol	81,91,92,134		2.2e ± 0		1.5g ± 0.1		0.1h ± 0		2.1f ± 0.1	1,2
18	1148	1147	Camphor	81, **95**, 108, 152	1961.3b ± 121.6	31.3g ± 0.2	1715.1b ± 2.3	30.8h ± 0	2599.8a ± 59.8	37.2e ± 0.1	1899.0b ± 399.2	31.5f ± 0.1	1,2,3
19	1174	1173	Borneol	79, 93, **95**, 110	166.1b ± 2.2	2.8g ± 0	171.7b ± 7.6	2.9f ± 0	279.7a ± 9.6	4.3e ± 0	164.1b ± 4.6	2.8f ± 0	1,2,3
20	1161	1180	Terpinen-4-ol	71, 86, **93**, 111	35.8a ± 1.7	0.7e ± 0	32.6ab ± 2.4	0.6f ± 0	28.9b ± 2.2	0.6f ± 0	37.0a ± 2.3	0.7e ± 0	1,2,3
21	1172	1190	p-Cymen-8-ol	43,91,135,150		0.1f ± 0		0.1f ± 0		0.1g ± 0		0.2e ± 0	1,2
22	1192	1198	α-Terpineol	59, 67, **93**, 121	44.2a ± 4.9	0.5g ± 0	43.3a ± 2.5	0.6f ± 0	13.9b ± 0.7	0.2h ± 0	51.0a ± 2.7	0.6e ± 0	1,2,3
23	1191	1201	(−)-Myrtenol	79, 91, 93, 119		0.1f ± 0		0.1f ± 0	8.3 ± 0.4	0.1e ± 0		0.1e ± 0	1,2,3
24	1223	1238	(*Z*)-Citral	79, 91, 119, 134		tr				0.1 ± 0		tr	1,2
25	1236	1249	Linalyl acetate	41, 69, **93**, 80	123.5c ± 3.5	1.5g ± 0	175.8a ± 1.9	2.3e ± 0			149.8b ± 1.4	2.1f ± 0.1	1,2,3
26	1232	1255	Geraniol	41, **69**, 93, 123	27.7a ± 1.1	0.4e ± 0	24.5a ± 2.3	0.4e ± 0	25.6a ± 0.4	0.4e ± 0	19.6a ± 5.9	0.4e ± 0	1,2,3
27	1285	1285	Bornyl acetate	79, **93**, 95, 121	40.3b ± 1.1	0.7g ± 0	50.6a ± 0.9	0.9f ± 0	50.7a ± 1.5	1.1e ± 0	39.0b ± 0.7	0.7g ± 0.1	1,2,3
28	1299	1290	Sabinyl acetate	81, 92, 119, 134		3.2e ± 0.1		3.0f ± 0		tr		2.9f ± 0.1	1,2
29	1322	1344	α-Terpinyl acetate	67, 68, **93**, 121	60.9b ± 0.5	1.2f ± 0	41.4c ± 0.2	0.8g ± 0	4.4d ± 0.3	0.1h ± 0	74.4a ± 0.2	1.6e ± 0	1,2,3
30	1360	1377	Geranyl acetate	41, 67, **69**, 93	9.7a ± 0.2	0.4e ± 0	9.3a ± 0.3	0.4f ± 0	8.6b ± 0.1	0.2g ± 0	9.1ab ± 0.2	0.5e ± 0	1,2,3
31	1412	1401	α-Gurjunene	91,105,161,204		0.1g ± 0		0.1f ± 0		0.1e ± 0		0.1h ± 0	1,2
32	1421	1419	(*E*)-β-Caryophyllene	**41**, 91, 93, 133	54.7c ± 0.9	0.7g ± 0	59.3b ± 1.7	0.9f ± 0	144.7a ± 0.5	2.3e ± 0	43.6d ± 1.2	0.7g ± 0	1,2,3
33	1454	1459	α-Humulene	80, **93**, 107, 204	10.1c ± 0.2	0.3g ± 0	11.9b ± 0.4	0.3f ± 0	23.2a ± 0.4	0.8e ± 0	8.9d ± 0.1	0.2g ± 0	1,2,3
34	1454	1476	Geranyl propionate	41, 69, 93, 120		0.4f ± 0		0.5e ± 0				0.4f ± 0	1,2
35	1472	1482	α-Curcumene	105, 119, 132, 145		0.1f ± 0		0.1f ± 0		0.3e ± 0		tr	1,2
36	1494	1496	α-Muurolene	91, 93, 119, 161		0.1f ± 0		0.1e ± 0		0.1e ± 0		0.1f ± 0	1,2
37	1507	1510	γ-Cadinene	91, 105, 119, 161		tr		0.1f ± 0		0.1e ± 0			1,2
38	1514	1517	δ-Cadinene	91, 119, 134, 161		0.1fg ± 0		0.1f ± 0		0.1e ± 0		0.1g ± 0	1,2
39	1575	1579	Caryophyllene oxide	41, 79, **91**, 109	8.9b ± 0.8	0.3f ± 0	9.5b ± 0.3	0.3f ± 0	17.2a ± 2	0.4e ± 0	10.3b ± 1.1	0.3f ± 0	1,2,3
40	1594	1593	Viridiflorol	43, 109, 161, 204		0.1h ± 0		0.1e ± 0		0.1f ± 0		0.1g ± 0	1,2
Oxygenated terpenes:													
Alcohol						8.3		8.2		6.5		8.8	
Ketone						31.3		30.8		37.2		31.5	
Aldehyde						0		0		0.1		0	
Ester						7.5		8		1.4		8.2	
Ether						24.5		26.1		22.1		25	
Monoterpene hydrocarbons						26.7		24.8		28.2		24.5	
Oxygenated monoterpenes						71.3		72.7		66.8		73.2	
Sesquiterpene hydrocarbons						1.2		1.7		3.8		1.2	
Oxygenated sesquiterpenes						0.4		0.4		0.5		0.4	
Total terpene hydrocarbons						27.9		26.5		32		25.7	
Total oxygenated terpenes						71.6		73.1		67.3		73.6	

^a^ Reference number for statistical PCA graphs; ^b^ Linear Retention Index from data bases NIST 08 & Wiley 7; ^c^ Linear Retention Index calculated from the homologous series of *n*-alkanes (C7–C30); ^d^ Quantitation ions are shown in bold. a, b, c, d: different letters in the same compound concentration mean statistically significant differences with *p* < 0.05. e, f, g, h: different letters in the same compound area mean statistically significant differences with *p* < 0.05. IM, Identification method: 1 = by LRI, 2 = by NIST 08 & Wiley 7, 3 = by comparison with pure compounds. tr, Traces (<0.1%).

**Table 2:** SlEO compositions compared with ISO standard.

Compound	ISO Standard ^a^		SlEO-1 (%)	SlEO-2 (%)	SlEO-3 (%)	SlEO-4 (%)
	Minimum (%)	Maximum (%)				
α-Pinene	4.0	11.0	7.0	6.2	7.2	6.2
Sabinene	0.1	3.5	1.8	1.8	2.4	1.7
Limonene	2.0	6.0	5.7	5.3	3.8	4.9
1,8-Cineole	10.0	30.0	31.3	32.9	29.4	31.7
Linalool	0.3	4.0	1.6	2.3	0.3	2.2
Camphor	11.0	36.0	40.4	39.3	50.3	40.3
Borneol	1.0	7.0	3.6	3.7	5.8	3.6
Terpinen-4-ol	-	2.0	0.9	0.8	0.8	0.9
Linalyl acetate	0.1	5.0	2.0	3.0	0.0	2.7
α-Terpinyl acetate	0.5	9.0	1.6	1.1	0.1	2.0
Sabinyl acetate	0.5	9.0	4.1	3.8	0.0	3.7

^a^ ISO standard 3526 for *Salvia lavandulifolia* [39].

**Table 3:** Enantiomeric ratios of SlEO compounds ^a^.

*t*_R_ (min)		Compound	SlEO-1		SlEO-2		SlEO-3		SlEO-4	
			(+)-X	(−)-X	(+)-X	(−)-X	(+)-X	(−)-X	(+)-X	(−)-X
(+)-X	(−)-X	(X)	(%)	(%)	(%)	(%)	(%)	(%)	(%)	(%)
7.79	7.52	α-Pinene	52	48	53	47	46	54	53	47
8.47	8.24	Camphene	26	74	27	73	32	68	39	61
8.89	9.16	β-Pinene	42	58	41	59	27	73	39	61
10.52	10.00	Limonene	81	19	86	14	63	37	87	13
14.28	14.51	Sabinene hydrate	>95	<5	>95	<5	>95	<5	>95	<5
15.73	15.57	Linalool	11	89	5	95	<5	>95	5	95
16.72	16.46	Camphor	76	24	80	20	79	21	77	23
18.02	18.18	Bornyl acetate	<5	>95	<5	>95	<5	>95	<5	>95
18.32	18.51	Terpinen-4-ol	69	31	81	19	51	49	80	20
20.10	19.76	α-Terpineol	85	15	>95	<5	>95	<5	>95	<5
20.15	19.58	Borneol	31	69	36	64	24	76	29	71
20.91	22.35	α-Terpinyl acetate	>95	<5	>95	<5	ND	ND	>95	<5
23.92	22.81	(*E*)-β-Caryophyllene	<5	>95	<5	>95	<5	>95	<5	>95

^a^ SD lower than ±5%.

**Table 4:** Antioxidant capacity of SlEOs.

SlEO	ORAC	DPPH	ABTS	RdP	ChP
	(mg TE/g SlEO)	(mg TE/g SlEO)	(mg TE/g SlEO)	(mg AAE/kg SlEO)	(mg EDTAE/g SlEO)
SlEO-1	101.1c ± 5.3	0.0c ± 0.0	0.3b ± 0.0	1.2c ± 0.1	3.9a ± 0.2
SlEO-2	136.1b ± 6.3	0.0bc ± 0.0	0.3b ± 0.0	1.7b ± 0.1	2.1c ± 0.1
SlEO-3	207.4a ± 10.0	0.1a ± 0.0	0.4a ± 0.0	1.9a ± 0.0	2.4bc ± 0.1
SlEO-4	125.0b ± 4.6	0.1b ± 0.0	0.5a ± 0.0	0.9d ± 0.0	2.6b ± 0.1

**Table 5:** Antioxidant capacity of SlEO compounds ^a^.

Compound	ORAC	DPPH	ABTS	RdP	ChP
	(µmol TE/mmol X)	(µmol TE/mmol X)	(µmol TE/mmol X)	(µmol AAE/mmol X)	(µmol EDTAE/mmol X)
α-Pinene	N/D	N/D	N/D	N/D	16.6 ± 1.1
Camphene	N/D	N/D	0.1 ± 0.0	0.5 ± 0.0	1.6 ± 0.1
β-Pinene	26.6 ± 1.6	N/D	0.1 ± 0.0	N/D	1.8 ± 0.1
Myrcene	N/D	N/D	N/D	N/D	2.5 ± 0.2
α-Terpinene	N/D	0.3 ± 0.0	4.0 ± 0.2	1.9 ± 0.1	62.2 ± 4.7
*p*-Cymene	N/D	N/D	0.1 ± 0.0	N/D	20.1 ± 1.6
Limonene	128.0 ± 10.8	N/D	0.6 ± 0.0	N/D	5.9 ± 0.4
1,8-Cineole	N/D	N/D	N/D	N/D	1.1 ± 0.1
γ-Terpinene	171.4 ± 10.3	0.3 ± 0.0	2.5 ± 0.1	0.3 ± 0.0	0.3 ± 0.0
Sabinene hydrate	N/D	N/D	0.5 ± 0.0	N/D	6.7 ± 0.6
Linalool	341.9 ± 18.5	N/D	0.1 ± 0.0	N/D	96.9 ± 5.8
Camphor	N/D	N/D	N/D	N/D	N/D
Borneol	N/D	N/D	N/D	N/D	N/D
Terpinen-4-ol	356.0 ± 13.6	N/D	0.3 ± 0.0	N/D	1.7 ± 0.1
α-Terpineol	310.0 ± 16.6	N/D	0.2 ± 0.0	N/D	4.9 ± 0.3
Linalyl acetate	207.1 ± 13.2	N/D	0.1 ± 0.0	N/D	27.4 ± 2.1
Bornyl acetate	N/D	N/D	N/D	N/D	N/D
β-Caryophyllene	394.7 ± 14.5	N/D	N/D	N/D	7.1 ± 0.5

^a^ N/D = Activity lower than 0.05 units at a maximum assay concentration of 100 mM.

**Table 6:** Antibacterial and antifungal capacities of SlEOs and main individual compounds.

SlEO/Compound	*Escherichia coli*		*Staphylococcus aureus*		*Candida albicans*	
	MIC (mg/mL)	MBC (mg/mL)	MIC (mg/mL)	MBC (mg/mL)	MIC (mg/mL)	MFC (mg/mL)
SlEO-1	9.0	9.0	4.5	4.5	2.2	4.5
SlEO-2	8.9	8.9	4.5	4.5	2.2	4.5
SlEO-3	8.9	8.9	4.5	4.5	2.2	4.5
SlEO-4	8.9	8.9	4.4	4.4	2.2	4.4
α-Pinene	0.5	1.0	2.1	>2.1	0.5	0.5
Camphene	>2.0	>2.0	>2.0	>2.0	>2.0	>2.0
Sabinene	>2.0	>2.0	>2.0	>2.0	>2.0	>2.0
β-Pinene	>2.0	>2.0	>2.0	>2.0	>2.0	>2.0
Myrcene	>2.1	>2.1	>2.1	>2.1	>2.1	>2.1
*p*-Cymene	1.0	2.0	>2.0	>2.0	0.5	0.5
Limonene	2.0	2.0	0.3	0.3	1.0	1.0
1,8-Cineole	>2.3	>2.3	>2.3	>2.3	>2.3	>2.3
Linalool	1.1	2.3	0.6	1.1	2.3	2.3
Camphor	>2.3	>2.3	>2.3	>2.3	>2.3	>2.3
Borneol	1.1	1.1	0.3	0.3	0.6	0.6
Terpinen-4-ol	2.3	2.3	1.1	2.3	>2.3	>2.3
α-Terpineol	2.4	2.4	0.6	1.1	>2.4	>2.4
Linalyl acetate	>3.0	>3.0	3.0	>3.0	>3.0	>3.0
Bornyl acetate	>2.9	>2.9	2.9	>2.9	>2.9	>2.9
β-Caryophyllene	>3.1	>3.1	>3.1	>3.1	>3.1	>3.1
α-Humulene	>3.0	>3.0	1.6	3.0	>3.0	>3.0
Streptomycin sulfate	1.0 × 10^−3^	1.0 × 10^−3^	1.0 × 10^−3^	1.0 × 10^−3^	NT	NT
Fluconazole	NT	NT	NT	NT	4.0 × 10^−3^	4.0 × 10^−3^

NT = Not tested.

## Supplementary Materials

Supplementary materials are available online.
