# Supplementary material for: Composition and Antioxidant, Antienzymatic and Antimicrobial Activities of Volatile Molecules from Spanish Salvia lavandulifolia (Vahl) Essential Oils
Source: Molecules. 2017 Aug 21;22(8):1382. doi: 10.3390/molecules22081382 (PMC6152226; doi:10.3390/molecules22081382)
Supplement: Supplementary file 1 [file molecules-22-01382-s001.pdf]

molecules

Composition and Antioxidant, Antienzymatic and Antimicrobial Activities of Volatile Molecules from Spanish *Salvia lavandulifolia* (Vahl) Essential Oils

Supplementary Materials

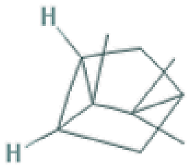

Tricyclene (**1**)

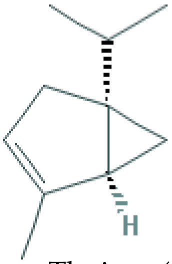

α-Thujene (**2**)

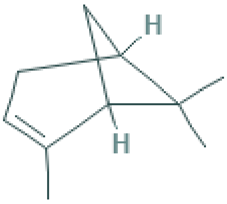

α-Pinene (**3**)

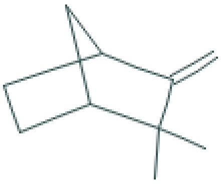

Camphene (**4**)

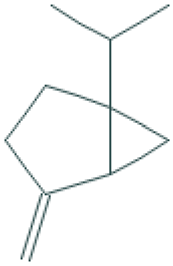

Sabinene (**5**)

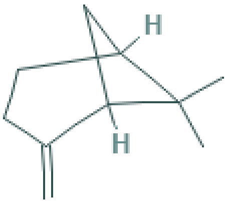

β-Pinene (**6**)

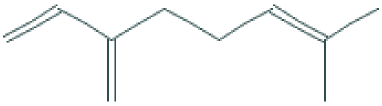

Myrcene (**7**)

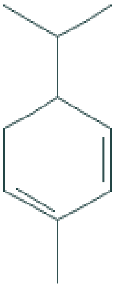

α-Pellandrene (**8**)

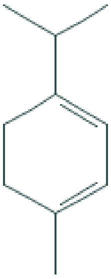

α-Terpinene (**9**)

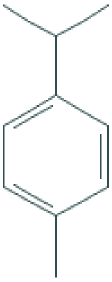

p-Cymene (**10**)

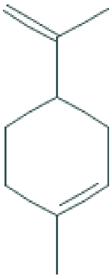

Limonene (**11**)

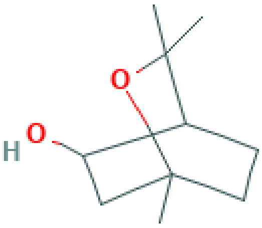

1,8-Cineole (**12**)

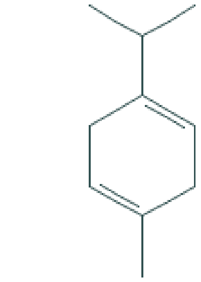

γ-Terpinene (**13**)

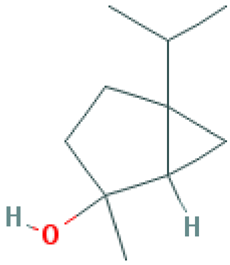

Sabinene hydrate (**14**)

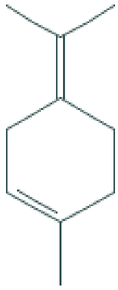

Terpinolene (**15**)

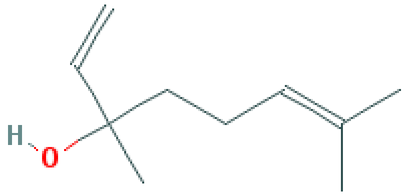

Linalool (**16**)

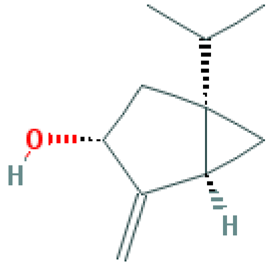

(−)-cis-Sabinol (**17**)

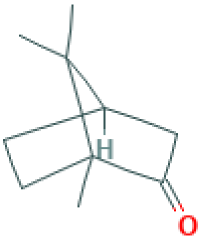

Camphor (**18**)

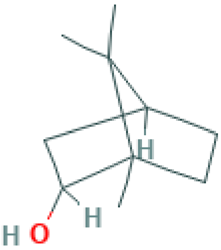

Borneol (**19**)

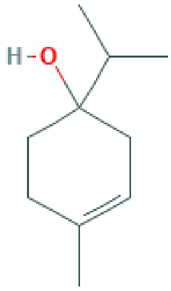

Terpinen-4-ol (**20**)

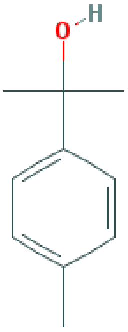

p-cymen-8-ol (**21**)

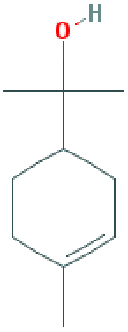

α-Terpineol (**22**)

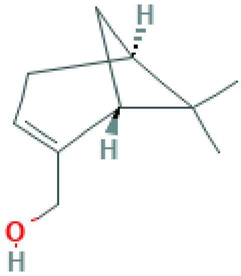

(−)-Myrtenol (**23**)

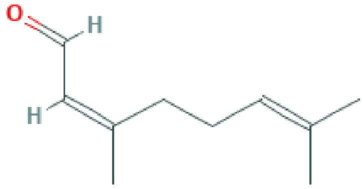

(*Z*)-Citral (**24**)

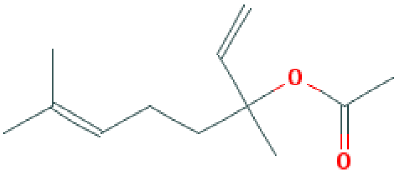

Linalyl acetate (**25**)

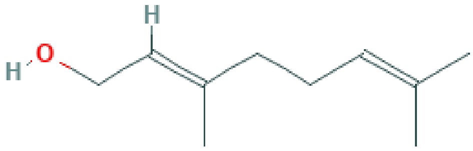

Geraniol (**26**)

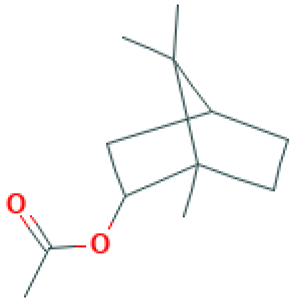

Bornyl acetate (**27**)

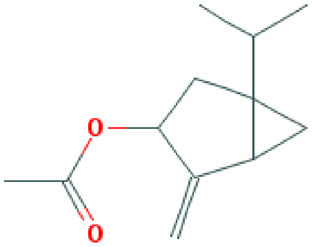

Sabinyl acetate (**28**)

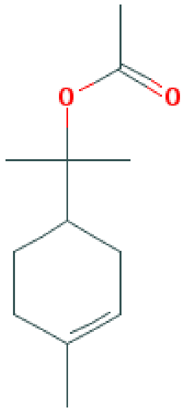

α-Terpinyl acetate (**29**)

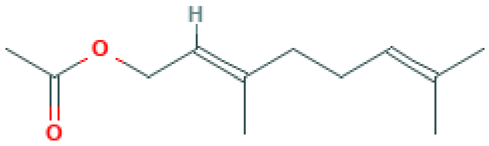

Geranyl acetate (**30**)

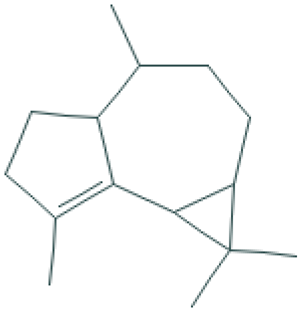

α-Gurjunene (**31**)

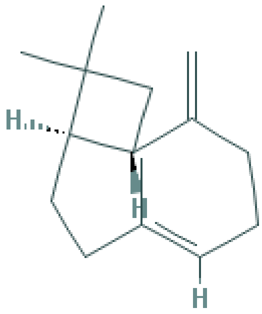

(*E*)-β-Caryophyllene (**32**)

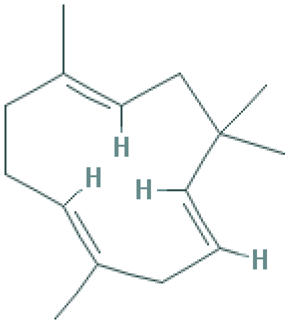

α-Humulene (**33**)

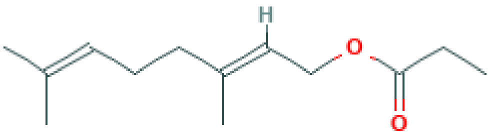

Geranyl propionate (**34**)

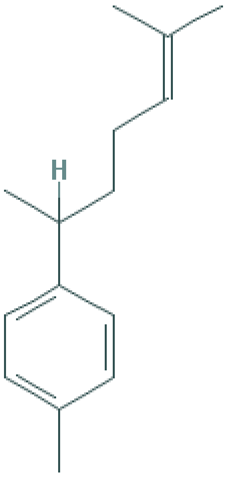

α-Curcurmene (**35**)

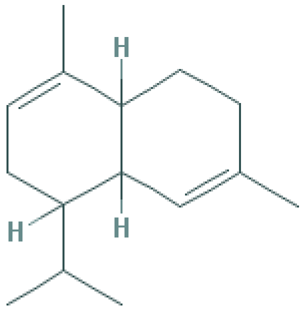

α-Muurolene (**36**)

molecules

MDPI

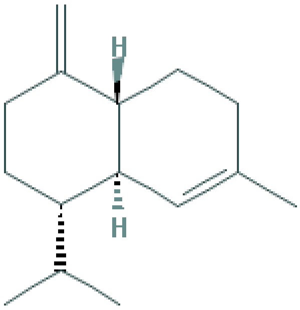

(+)-γ-Cadinene (**37**)

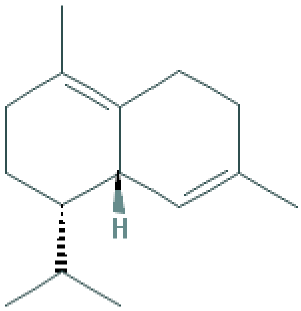

(+)-δ-Cadinene (**38**)

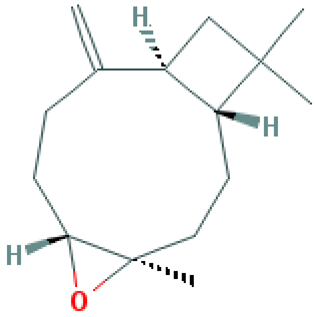

Caryophyllene oxide (**39**)

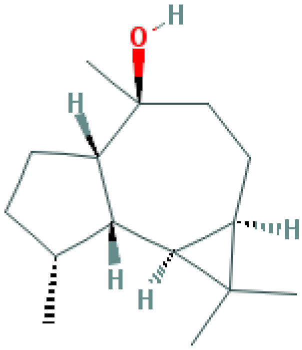

Viridiflorol (**40**)

Figure S1: Structures of compounds in the EOs.

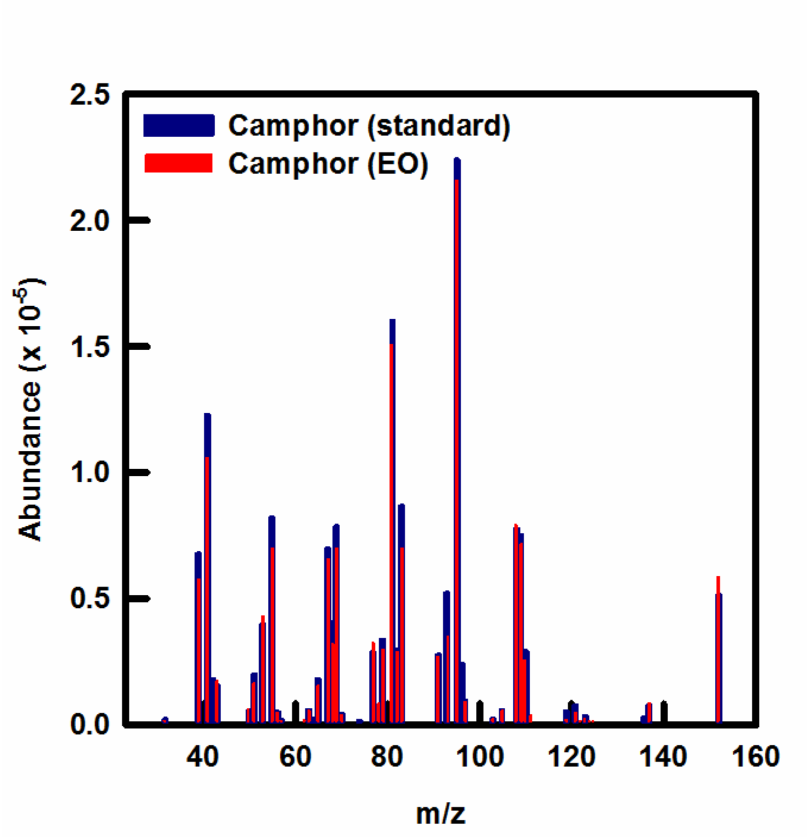

Figure S2: Mass spectra of camphor. Comparison between mass spectra of commercial and natural EO compound.

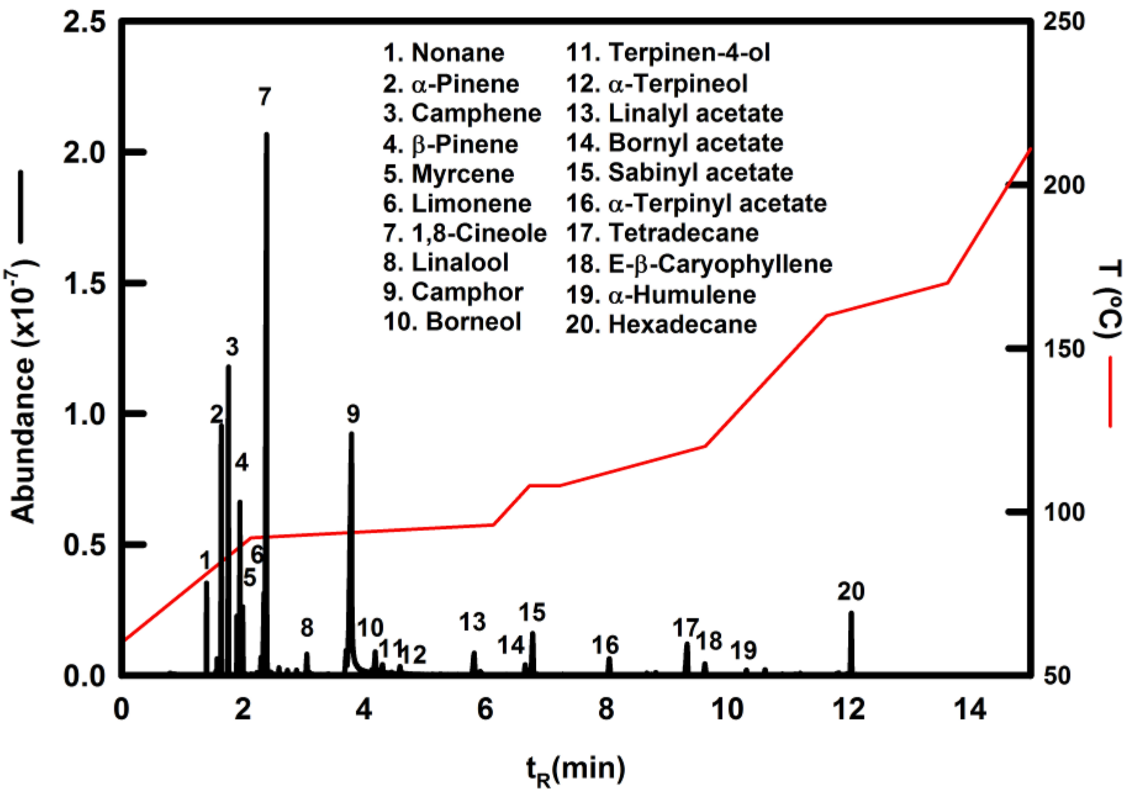

Figure S3: FGC-MS chromatogram of SlEO-1. Main compounds are identified with different numbers.

molecules

MDPI

Table S1: Calibration curve parameters to determine the absolute volatile concentration of SlEOs.

Compound	Calibration Curve[a]	R^2	Calibration Range (mmol/L)	RSD (%)	LOD (mmol/L)	LOQ (mmol/L)	Standard source	Product Reference
Nonane	Internal standard (MS 43[b], 57, 71, 85)						SAFC	442694
(−)α-Pinene	y = 1.272x − 0.008	0.999	10.06-0.25	0.97	0.025	0.076	Fluka	80599
(+)Camphene	y = 0.728x + 0.001	0.999	11.09-0.28	0.56	0.054	0.165	SAFC	w222909
Sabinene	y = -0.796x + 0.022	0.999	9.78-0.61	1.08	0.091	0.277	Extrasynthese	5062 S
(−)β-Pinene	y = 1.053x − 0.010	1.000	10.29-0.26	1.26	0.026	0.078	Fluka	80609
Myrcene	y = 0.675x − 0.011	0.999	8.97-0.22	1.41	0.018	0.056	Fluka	64643
(−)Phellandrene	y = 0.528x − 0.008	0.999	9.52-0.60	1.60	0.106	0.322	Aldrich	bcbb8659
α-Terpinene	y = 0.591x − 0.024	0.999	15.75-0.59	1.27	0.149	0.453	Aldrich	86473
p-Cymene	y = 2.709x − 0.003	0.999	9.95-0.25	1.08	0.010	0.030	Aldrich	c121452
(+)Limonene	y = 1.308x − 0.026	0.999	9.55-0.24	0.98	0.039	0.120	Fluka	62118
1,8-Cineole	y = 0.523x − 0.008	1.000	9.55-0.60	1.55	0.164	0.497	SAFC	w246506
γ-Terpinene	y = 0.906x − 0.018	0.999	9.87-0.25	1.04	0.020	0.062	Aldrich	223190
Sabinene hydrate	y = 0.562x − 0.013	0.999	9.66-0.60	3.17	0.102	0.309	FLUKA	96573
Terpinolene	y = 0.618x − 0.021	0.999	15.34-0.92	0.69	0.230	0.699	SAFC	W304603
(−)Linalool	y = 0.471x − 0.009	0.999	8.80-0.55	3.13	0.102	0.309	Fluka	74856
Tetradecane	Internal standard (MS 43, 57[b], 71, 85)						SAFC	442708
(+)Camphor	y = 0.090x − 0.007	0.999	9.64-0.96	0.86	0.241	0.733	Alfa Aesar	A10708
(−)Borneol	y = 0.154x − 0.002	0.999	8.99-0.90	1.93	0.196	0.595	Alfa Aesar	A12684
(−)Terpinen-4-ol	y = 0.285x − 0.009	0.999	9.57-0.96	3.59	0.239	0.727	Aldrich	11584
(+)α-Terpineol	y = 0.169x	0.998	9.87-0.99	1.97	0.247	0.750	Fluka	83073
R-(−)-Myrtenol	y = 0.183x + 0.002	0.999	16.87-2.53	2.62	0.440	1.337	SAFC	S37589-149
Linalyl acetate	y = 0.179x + 0.005	0.993	11.97-1.80	1.93	0.449	1.365	SAFC	w263605
Geraniol	y = 0.221x − 0.006	1.000	15.04-0.90	0.61	0.295	0.896	SAFC	w250716
(−)Bornyl acetate	y = 0.355x − 0.001	0.999	13.59-0.82	1.65	0.204	0.620	FLUKA	45855
(+)α-Terpinyl acetate	y = 0.296x	1.000	11.95-1.79	0.99	0.243	0.740	Aldrich	1439212
Geranyl acetate	y = 0.391x − 0.006	0.999	12.37-0.46	0.17	0.116	0.353	Aldrich	173495
(−)*E*-β-Caryophyllene	y = 0.094x + 0.003	0.999	6.35-1.09	2.49	0.360	1.093	Sigma	22075
α-Humulene	y = 0.416x − 0.011	0.999	6.35-0.63	1.85	0.154	0.469	Aldrich	53675
Hexadecane	Internal standard (MS 41, 43, 57[b], 71)						Fluka	52209
Caryophyllene oxide	y = 0.067x + 0.008	0.999	17.66-6.62	2.23	1.855	5.638	SAFC	w509647

[a] Response ratio vs. Concentration ratio, internal standard correction applied. Each internal standard is reference compound for the analytes that follow.[b] Quantifier ion. LOD = Limit of detection (signal/noise = 3). LOQ = Limit of quantitation (signal/noise = 10)

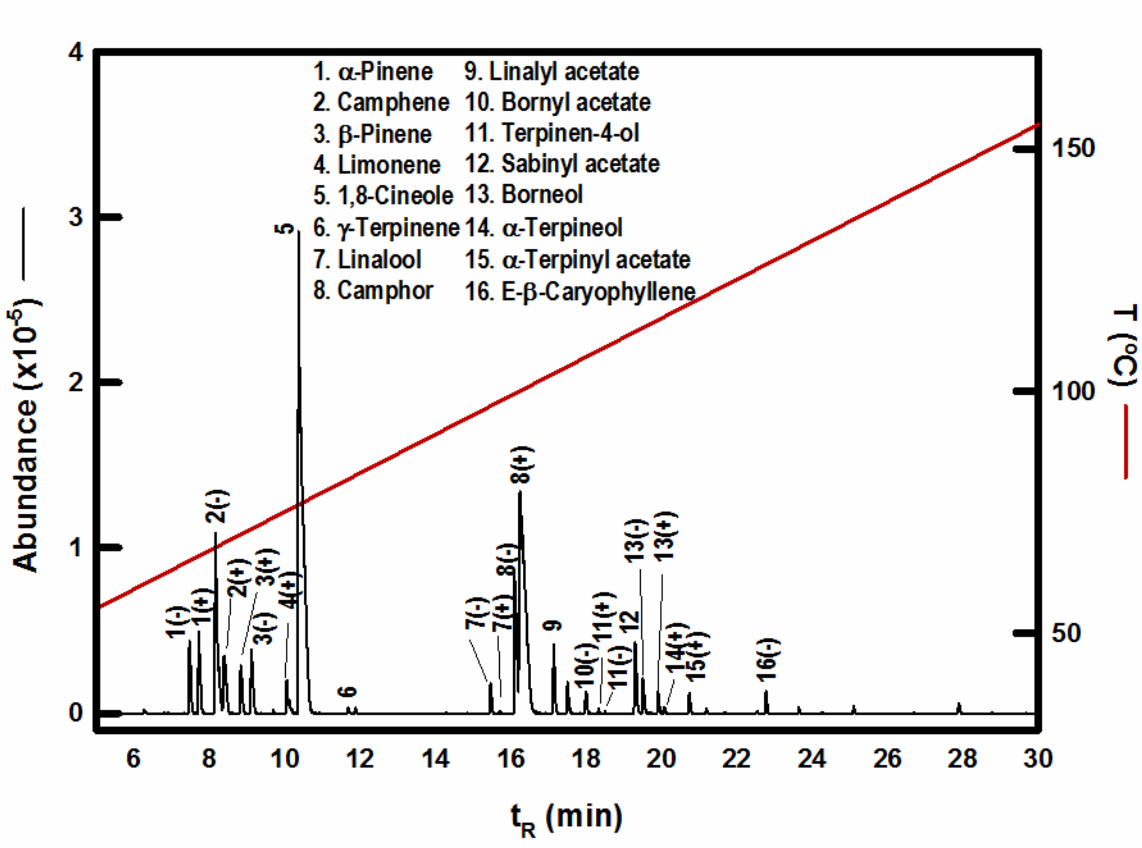

Figure S4: Enantioselective Chromatogram of SlEO-2. The dextrorotatory (+) and levorotatory (−) main enantiomers are identified with different numbers.

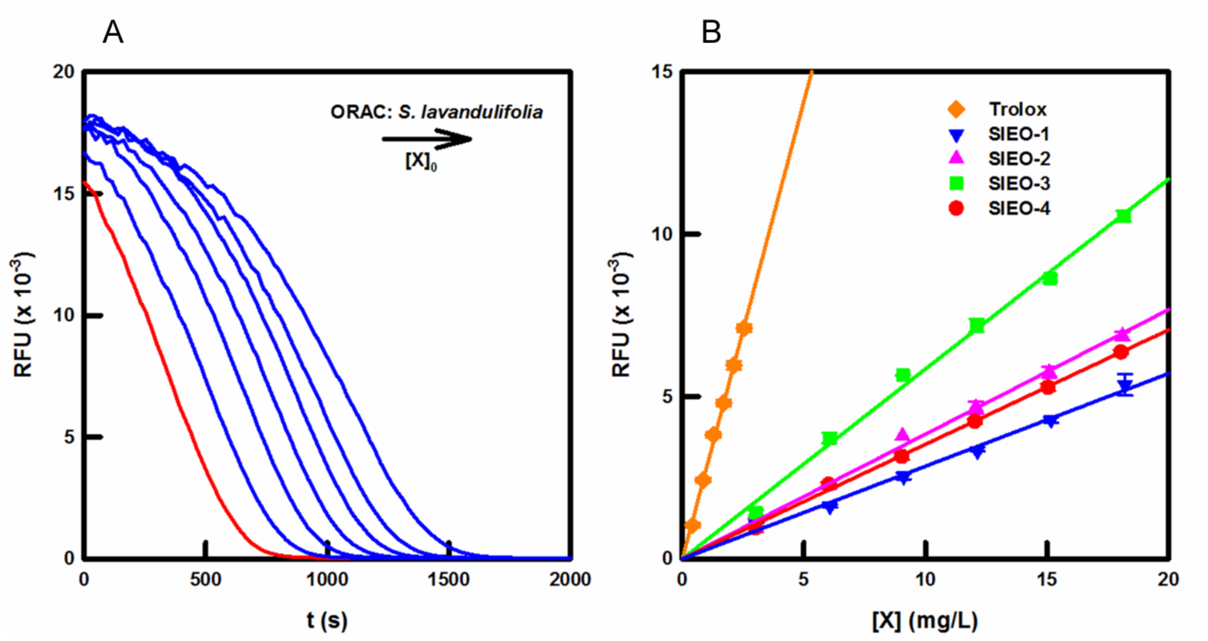

Figure S5: Antioxidant capacity of SlEO using ORAC method. (A) Fluorescence curves with different concentrations of SlEO-3. (B) Linear regressions of the area under curve (AUC) with the increase in SlEO concentration.

molecules

MDPI

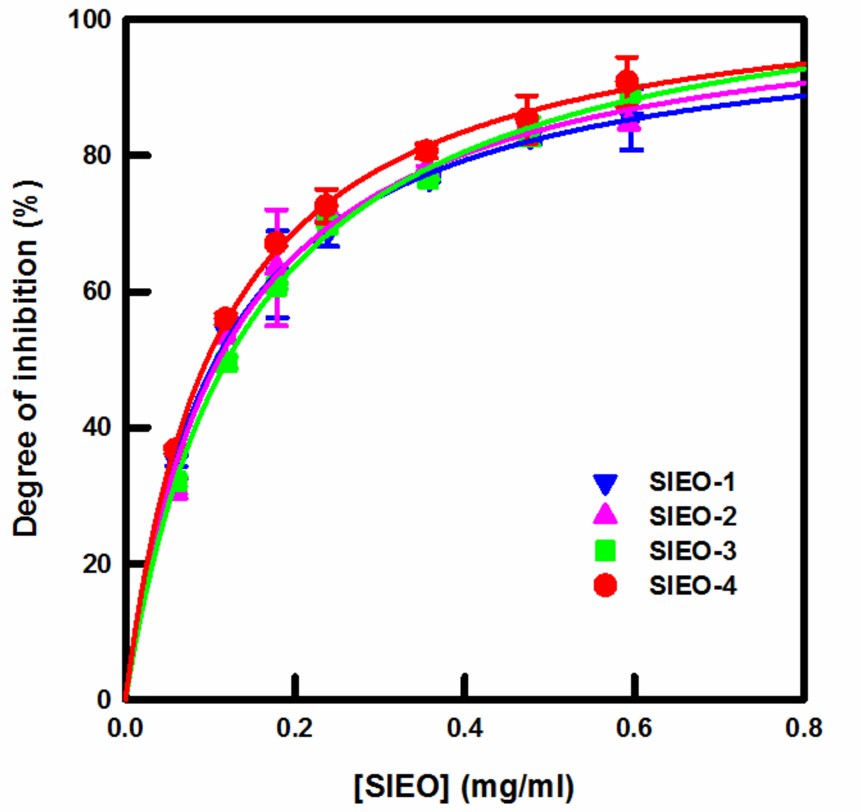

Figure S6: Determination of IC_{50} of SlEOs. Kinetic analysis of enzyme inhibition data with non-linear regression.
